# Supplementary material for: Male-Type Depression Mediates the Relationship between Avoidant Coping and Suicidal Ideation in Men
Source: Int J Environ Res Public Health. 2022 Aug 31;19(17):10874. doi: 10.3390/ijerph191710874 (PMC9517898; doi:10.3390/ijerph191710874)
Supplement: Supplementary file 1 [file ijerph-19-10874-s001.zip › ijerph-1873692-supplementary.pdf]

Supplement S1: Demographic questions

Q: What is your age in years? Please just write the number.
Response option(s): [Numeric text]

Q: How would you describe your current place of residence?

Response option(s):

- ☐ Metropolitan
- ☐ Regional
- ☐ Rural or remote

Q: What state/territory do you live in?

Response option(s):

- ☐ Western Australia
- ☐ Queensland
- ☐ Northern Territory
- ☐ South Australia
- ☐ New South Wales
- ☐ Victoria
- ☐ Tasmania
- ☐ Australian Capital Territory
- ☐ Jervis Bay Territory

Q: What is your postcode?

Response option(s): [Numeric text]

Q: What is your current employment status?

Response option(s):

- ☐ Employed full time
- ☐ Employed part time
- ☐ Employed casually
- ☐ Unemployed, looking for work
- ☐ Unemployed, not looking for work
- ☐ Retired
- ☐ Student

Q: What is your highest level of completed education?

Response option(s):

- ☐ Some high school
- ☐ Trade/certificate/diploma
- ☐ High school
- ☐ Undergraduate degree
- ☐ Postgraduate degree

Q: What is your current income range? (Australian dollars)

Response option(s):

- ☐ $0 - $49,999 per year (1)
- ☐ $50,000 - $99,999 per year (2)
- ☐ $100,000 - $149,999 per year (3)
- ☐ $150,000 - $199,999 per year (4)
- ☐ $200,000 + per year (5)

Q: What is your gender?

Response option(s):

- ☐ Male
- ☐ Another gender: [Free text response]

Q: Do you identify as transgender?

Response option(s):

- ☐ Yes
- ☐ No

Q: How would you best describe your sexual orientation?

Response option(s):

- ☐ Straight
- ☐ Gay
- ☐ Bisexual
- ☐ Other: [Free text response]

Q: What is your current relationship status?

Response option(s):

- ☐ Single/never married
- ☐ Partnered
- ☐ Married / de facto
- ☐ Single, separated / divorced
- ☐ Widowed
- ☐ Other: [Free text response]

Q: What category best describes your current living situation?

Response option(s):

- ☐ Single person living alone
- ☐ Non-related adults sharing a home
- ☐ Couple living with no children
- ☐ Couple with dependent children living at home (dependent defined as financially or physically dependent)
- ☐ Single parent with dependent children living at home
- ☐ Single person living with extended family (with or without children)
- ☐ Couple living with extended family (with or without children)
- ☐ Other, please specify: [Free text response]

Q: Do you identify as Aboriginal or Torres Strait Islander?

Response option(s):

- ☐ No
- ☐ Yes - Aboriginal
- ☐ Yes - Torres Strait Islander
- ☐ Both Aboriginal and Torres Strait Islander

Q: How would you describe your cultural/ethnic background?

Response option(s): [Free text response]

Q: What country were you born in?

Response option(s):

- ☐ Australia
- ☐ Somewhere else: [Free text response]

Supplement S2: COVID-19 pandemic stressors questions

Q: To what extent has the COVID-19 pandemic put financial stress on you?

Response option(s):

1. No Stress
2. A little stress
3. Moderate Stress
4. Considerable Stress
5. Extreme Stress

Q: How have government COVID-19 restrictions affected your mental health?

Response option(s):

1. Very positively
2. Somewhat positively
3. Not at all
4. Somewhat negatively
5. Very negatively

Supplement S3: Mediation analysis without covariates

Supplementary Table S1. Mediation model assessing the mediating role of male-type depression in the relationship between avoidant coping and suicidal/self-harm ideation (*n*=606)

	β	***B* (*SE*)**	**99% CI**	**T**	***p***
Direct effect of AC on SI/SHI	0.27	.06 (.01)	.03-08	5.831	**<.001**
Total effect of AC on SI/SHI	0.44	.09 (.01)	.07-.11	12.129	**<.001**
Indirect effect of AC on SI/SHI (via MDRS-7 score)	0.18	.03 (.01)	.02-.06		

Note. Boldface text indicates statistically significant values at $p<.01$, AC = avoidant coping, SI/SHI = suicidal ideation/self-harm ideation, β = standardised coefficient, *B* = unstandardised coefficient, *SE* = standard error, CI = confidence interval.
